# Delayed diagnosis of ventricular lead malposition in the left ventricle via a patent foramen ovale

**DOI:** 10.1093/ehjcr/ytaf485

**Published:** 2025-10-03

**Authors:** Georgia Goranitou, Nikitas Saroukos, Eleni Kalkandi, Paraskevi Georgiou, Skevos Sideris

**Affiliations:** 1st Department of Cardiology, General Hospital of Nikea Piraeus ‘St Panteleimon’, 3 Dimitrios Mantouvalos Str, 18454 Nikea Pireaus, Greece; 1st Department of Cardiology, General Hospital of Nikea Piraeus ‘St Panteleimon’, 3 Dimitrios Mantouvalos Str, 18454 Nikea Pireaus, Greece; 1st Department of Cardiology, General Hospital of Nikea Piraeus ‘St Panteleimon’, 3 Dimitrios Mantouvalos Str, 18454 Nikea Pireaus, Greece; 1st Department of Cardiology, General Hospital of Nikea Piraeus ‘St Panteleimon’, 3 Dimitrios Mantouvalos Str, 18454 Nikea Pireaus, Greece; 2nd Department of Cardiology, Hippokrateion General Hospital of Athens, 114 Vasilissis Sofias Avenue, 11527 Athens, Greece

## Case description

**Figure 1:**
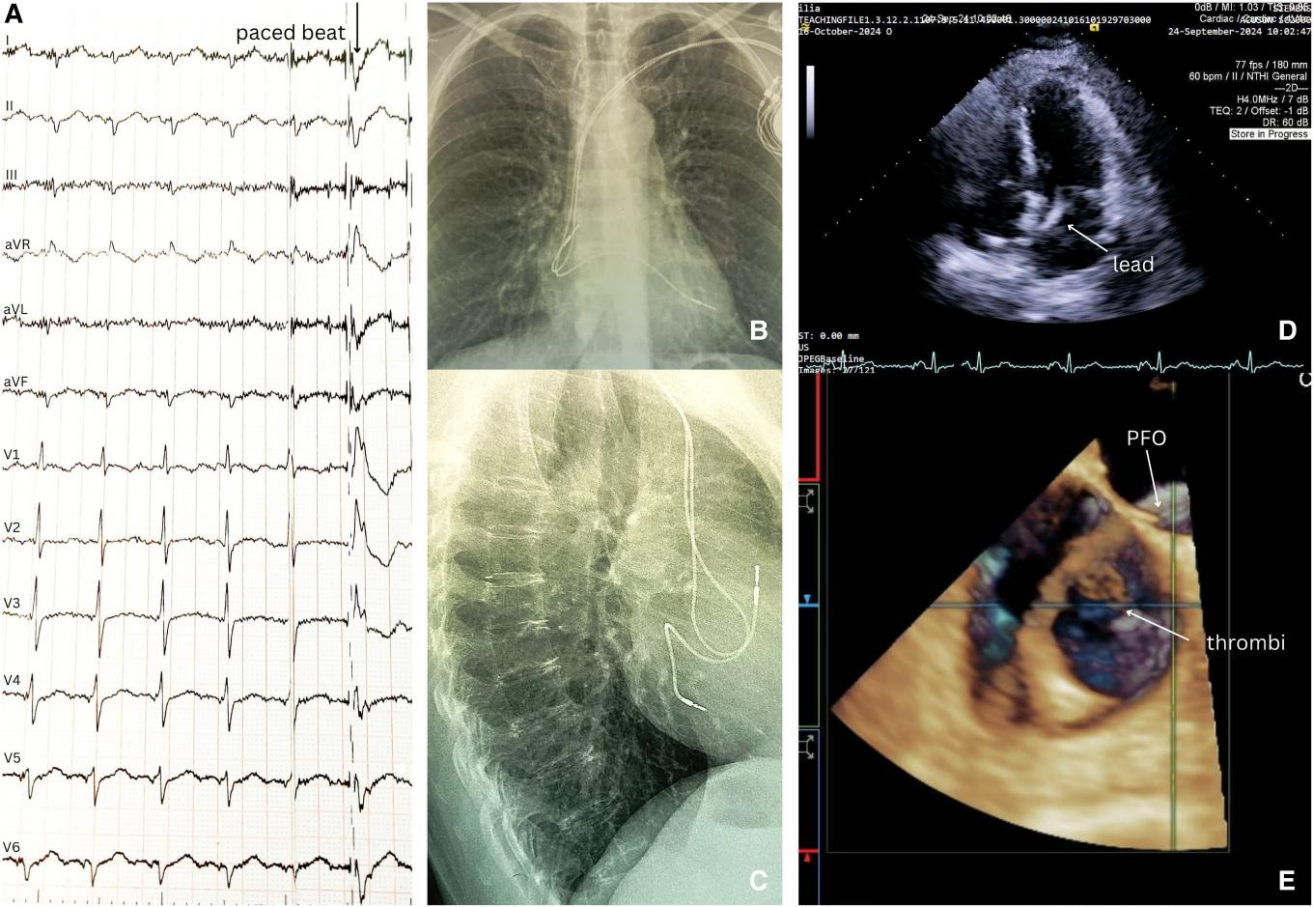
Diagnostic findings of ventricular lead malposition in the left ventricle. (*A*) Twelve-lead electrocardiogram: sinus rhythm with a ventricular paced beat (arrow) showing right bundle branch block morphology with late precordial transition at lead V_5_. (*B*) Posteroanterior chest X-ray: abnormal superior-leftward ventricular lead course. (*C*) Lateral chest X-ray: posterior ventricular lead trajectory, suggesting LV position. (*D*) 2D transthoracic echocardiography (apical four-chamber view): ventricular lead (arrow) traversing interatrial septum, crossing mitral valve, and anchored in LV inferoseptum. (*E*) 3D transoesophageal echocardiography (bicaval view): ventricular lead crossing a patent foramen ovale (arrow) with attached thrombi (arrow).

A 55-year-old woman with systemic lupus erythematosus and a dual-chamber pacemaker (implanted 6 years earlier for sick sinus syndrome) presented with dizziness and gait unsteadiness. Neurological examination and brain CT angiography were unremarkable. The patient was in sinus rhythm. Device interrogation showed normal pacemaker function, but the 12-lead paced electrocardiogrram exhibited right bundle branch block (RBBB) morphology with delayed precordial transition at lead V_5_, suggesting ventricular lead malposition in the left ventricle (LV)^1,2^ (*Figure 1A*).

The posteroanterior chest radiography (PA X-ray) revealed an abnormally high and leftward position of the ventricular lead, while the lateral view showed a posterior trajectory (*Figure 1B* and *C*). Transthoracic echocardiography (TTE) demonstrated the ventricular lead traversing the interatrial septum, crossing the mitral valve, and anchoring in the LV inferoseptal wall (*Figure 1D*). Transoesophageal echocardiography (TEE) confirmed passage through a patent foramen ovale with two adherent thrombi (*Figure 1D*). Positron emission tomography/computed tomography excluded infection.

The patient was started on acenocoumarol (target INR 2.5–3.0). After 4 weeks, both atrial and ventricular leads were percutaneously extracted, and a new pacemaker was implanted contralaterally without complications. Anticoagulation continued for 1-month post-procedure. At 4-month follow-up, she remained asymptomatic.

This case illustrates the need for (i) heightened awareness of delayed lead malposition presentations, (ii) systematic evaluation of paced QRS morphology (e.g. RBBB pattern) integrated with multimodal imaging (X-ray, TTE, TEE) in pacemaker recipients with neurologic symptoms, and (iii) immediate intervention upon stroke identification to mitigate further thromboembolic risk.

**Consent:** Written informed consent was obtained from the patient for publication of this case report and any accompanying images, in line with the Committee on Publication Ethics (COPE) guidelines.

**Funding:** No funding was received for this manuscript.

## Data Availability

All data underlying this article are included within the article itself. No additional data are available.
